# Chemicals released by male sea cucumber mediate aggregation and spawning behaviours

**DOI:** 10.1038/s41598-017-18655-6

**Published:** 2018-01-10

**Authors:** Nathalie Marquet, Peter C. Hubbard, José P. da Silva, João Afonso, Adelino V. M. Canário

**Affiliations:** CCMAR-Centre of Marine Sciences, Universidade do Algarve, Campus de Gambelas, 8005-139 Faro, Portugal

## Abstract

The importance of chemical communication in reproduction has been demonstrated in many marine broadcast spawners. However, little is known about the use of chemical communication by echinoderms, the nature of the compounds involved and their mechanism(s) of action. Here, the hypothesis that the sea cucumber *Holothuria arguinensis* uses chemical communication for aggregation and spawning was tested. Water conditioned by males, but not females, attracted both males and females; gonad homogenates and coelomic fluid had no effect on attraction. Male spawning water, but not female spawning water, stimulated males and females to release their gametes; the spermatozoa alone did not induce spawning. *H*. *arguinensis* male spawning water also induced spawning in the phylogenetically related *H*. *mammata*. This indicates that males release pheromones together with their gametes that induce spawning in conspecifics and possibly sympatric species. Finally, the male pheromone seems to be a mixture with at least one labile compound (biological activity is lost after four hours at ambient temperature) possibly including phosphatidylcholines. The identification of pheromones in sea cucumbers offers a new ecological perspective and may have practical applications for their aquaculture.

## Introduction

Broadcast spawning is considered the most ancient and widespread mode of reproduction in marine invertebrates^1,2^. The main disadvantage of this reproductive mode is the rapid dispersal of gametes in the environment, which can reduce fertilization rates and subsequent larval production^3,4^. To counteract this, organisms have adopted different behavioural strategies including breeding aggregation and synchronization of reproductive activities^5–7^.

While environmental factors such as temperature, photoperiod, lunar periodicity and tidal cycles seem to promote invertebrate aggregative behaviour and spawning possibly by stimulating reproductive processes^8^, chemical communication plays a determinant role in the fine tuning of these processes in several marine broadcasters such as polychaetes, crustaceans, molluscs and echinoderms^9–14^.

Although aggregations are regularly found in echinoderms, what brings individuals to group together is still poorly understood. Aggregative behaviours are understood to facilitate gametogenesis and spawning through inter-individual chemical exchange and by increasing the probability of gamete encounter^15–19^. Indeed, field observations show that grouped animals, irrespective of the sex ratio, are riper than solitary individuals^20^.

Among echinoderms, the existence of pheromones synchronizing gamete release among individuals was first suggested by Beach, *et al*. in the starfish *Acanthaster planci* ^21^. However, evidence is largely circumstantial and little is known about the chemical nature, origin and mechanisms of action of the putative pheromones^22^. Echinoderm males generally start to spawn before females, suggesting that spermatozoa and/or chemicals released with the sperm stimulate the females^23–27^. Sperm suspensions have been experimentally tested on the spawning behaviour in starfish^28,29^ and sea urchins^30,31^. However, the reciprocal effects of male and female gametes have rarely been experimented, and the origin and identity of the chemicals involved have never been established in any echinoderms.

Kato, *et al*.^32^ induced spawning of mature sea cucumbers by injection of a gonadatropic neural peptide (NGIWYamide), extracted from buccal ring nerves, into the coelomic cavity. This technique, originally developed in starfish^33^, proved ineffective when the radial nerve extract was simply added to the water, rejecting a pheromonal role^34^. In contrast, intra-coelomic injections of perivisceral coelomic fluid (PCF) from spawning individuals successfully induced spawning directly and when added to the water, indicating that the coelomic fluid may contain a pheromone^35^.

The commercial demand for sea cucumbers has led to over-exploitation and severe depletion or disappearance in many regions worldwide and aquaculture programs are being developed to sustain and to enhance wild populations^36^. The sea cucumber *Holothuria arguinensis* is a recent fisheries target and the first sea cucumber species to be reared in captivity in Europe^37–39^. A better understanding of the chemical factors influencing the reproductive biology of this broadcast summer-autumn spawner could give valuable insights to improve the management of species reared in captivity. Thermal shock remains the most common used method to stimulate spawning in their aquaculture^40–42^. However, this method gives inconsistent and variable results according to the protocol and species used^43,44^; therefore, the identification of spawning pheromones may provide a promising alternative. Knowledge of the pheromonal chemicals could also help to control invasive species, which represent a major threat to biodiversity and cause significant damage to worldwide economy^45^. A typical case is found in the Mediterranean Sea where many Indo-Pacific species, including sea cucumbers, have invaded the area through the Suez Canal^46,47^.

Here, the hypothesis that the sea cucumber *H*. *arguinensis* uses chemical communication for aggregation and spawning was tested. We show that *H*. *arguinensis* males and females are attracted by male conditioned water but not by female conditioned water, gonad homogenates or coelomic fluid. Furthermore, male spawning water (but not female spawning water or sperm), induces spawning in males and females. The *H*. *arguinensis* spawning water also induces spawning in the related *H*. *mammata*. Finally, we attempt to identify the pheromone and results indicate they are a mixture containing at least a labile compound and possibly phosphatidylcholines. Altogether, our results provide a novel perspective into sea cucumber aggregative and spawning behaviour with practical applications in ecology and aquaculture.

## Material and Methods

### Ethics statement

Sea cucumbers, *Holothuria arguinensis*, Koehler & Vaney, 1906, and *Holothuria mammata*, Grube, 1840, were collected and handled in agreement with the license issued by ICNF, The Institute for Nature and Forest Conservancy of Portugal (License N°635/2015/CAPT, N°95/2016/CAPT, N°490/2016/CAPT). The species are not endangered or protected, and those used in experiments were released back into their natural environment.

### Collection, gonadal biopsy and maintenance of specimens

*H*. *arguinensis* longer than 210 mm, i.e. adults according to previous analysis of the sexual maturity in this species^39^, were collected from southern Portugal: during late spring 2015 from the Ria Formosa (37°00′35.02″N; 7°59′46.10″O) for the aggregation behaviour assay and during summers of 2015 and 2016 from Sagres (37°00′44.78″N; 8°55′49.51″O) for the spawning experiment. Adult *H*. *mammata* (200–250 mm), a sister species to *H*. *arguinensis* ^48^, were collected from the Ria Formosa. The sex and the maturity stage of the sea cucumbers were determined according to Marquet *et al*.^39^ by observation under a light microscope (Leica DM2000) of a gonadal biopsy taken from a small incision on the dorsal side of the animal previously anesthetized in 5% MgCl_2_^9^. All experiments were performed at least one week after the biopsy to allow recovery (all animals recovered without any obvious signs of infection or permanent damage). Females and males were kept in separate tanks (1.2 × 1.0 × 0.6 m) in natural sea water and fed four times a week with sediment collected from their natural environment.

### Y-maze tests of attraction

**Figure 1:**
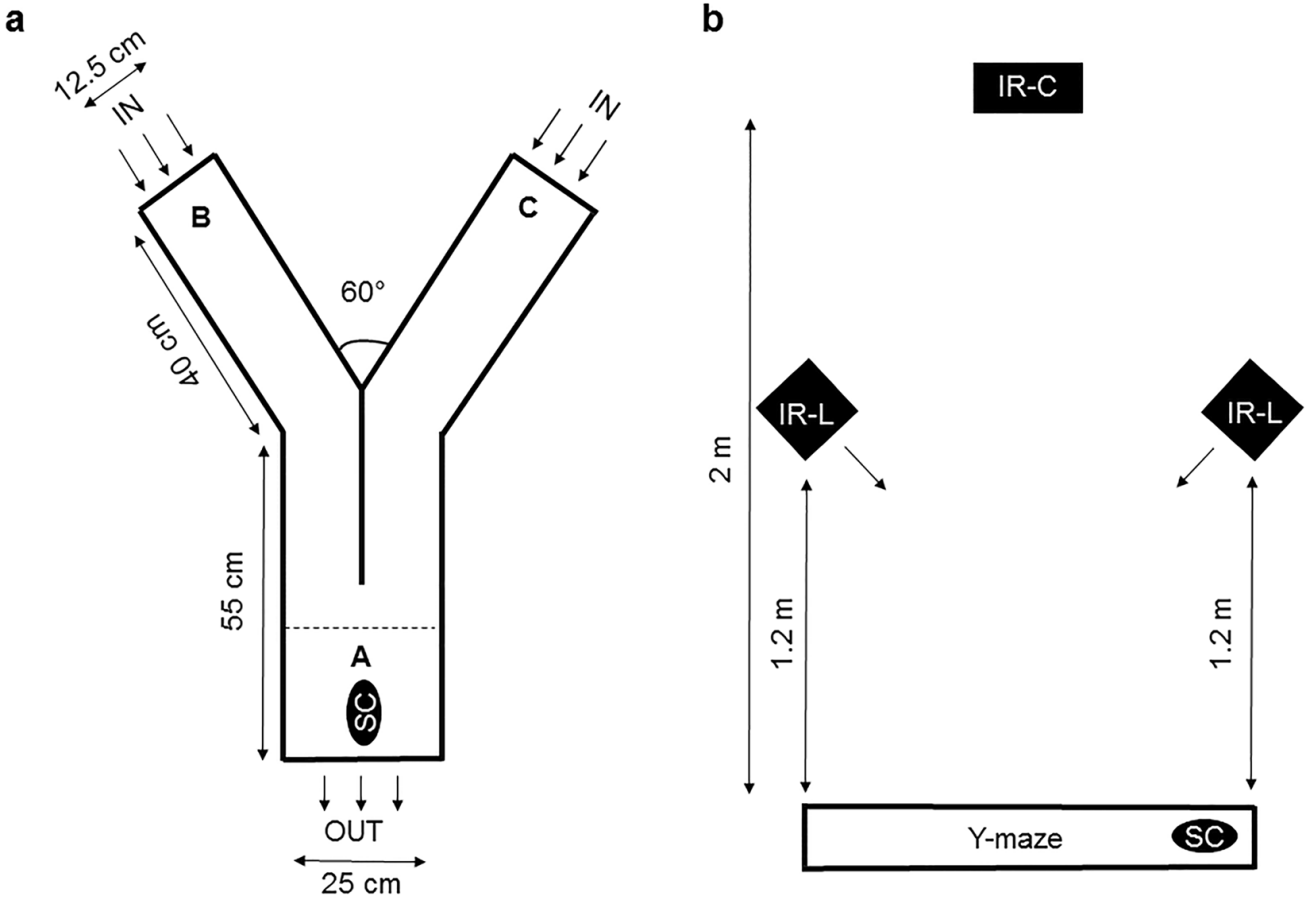
Schematic representation of the experimental setup used in the attraction experiment. (**a**) Overhead view showing the entry area A, the stimulus and control sides B or C. (**b**) Side view showing the position of the camera IR-C and light source IR-L. SC indicates the initial positioning of the test sea cucumber.

To test the capacity of water-borne stimuli to attract conspecifics, a glass Y-maze (30 cm height, 3 mm thick) was used with a stem of 55 cm long and 25 cm wide separating into two arms of 40 cm long and 12.5 cm wide at the end of which stimuli were added (Fig. 1a). Water inflow was 700 ml/min in each arm and drained out of the maze through two holes (2 cm diameter) connected to a standpipe which maintained the water height (10 cm). Tests of the maze plume dynamics using food colouring (Brilliant Blue FCF, E133) delivered to both arms revealed by visual inspection small-scale turbulence within the arms, but little mixing between water of the two arms in the stem section. Data collected was based on first entry and time spent in either of the two arms where there was no mixing. The Y-maze was surmounted at 2 m height by an infrared video camera equipped with infrared filter (ICD-49E, Ikegami Tsushinki, Japan) and at 1.2 m height by two automated infrared light sources (IR-56, Microlight, Russia) oriented diagonally with respect to the bottom of the Y-maze (Fig. 1b). The videos were stored in AVI files in a hard drive and displayed with Everfocus Player Application (EFPlayer v 1.0.6.4.).

Experiments were carried out over four hours at night, when this species is more active. All animals used in this experiment were at stage III to IV - mature oocytes or spermatozoa filled the gonadal tubules almost completely^39^. Test animals were placed at the entry area A of the Y-maze (Fig. 1a) and given a choice of control seawater and seawater containing the stimulus delivered to each arm (B and C) at a rate of 700 ml/min. The stimulus side was alternated between successive tests to eliminate arm preference. The stimuli tested were: (1) conspecific-conditioned water (CCW), (2) gonad homogenates (ovary and testis), and (3) coelomic fluid (CF). To produce conspecific-conditioned water, two individuals of known sex were placed in an aquarium (30 × 20 × 20 cm) from which water flowed by gravity to one of the Y-maze arms. Separate pools of five testes and five ovaries (total of 120 g each) were homogenised from fresh gonad with a mortar and a pestle and filtered (100 µm pore size) to remove large particles. Coelomic fluid (10 ml) was collected from separate pools of five males and five females using a sterile needle inserted in the body wall of the animals and withdrawn by gravity. Gonad homogenates and coelomic fluid pools were frozen at −20 °C after collection. The day of the experiment they were thawed and diluted in 1200 ml of seawater and injected in the maze with a peristaltic pump at a rate of 10 ml/min during the first two hours of the experiments, with the seawater inflow of 700 ml/min.

At least 10 males and 10 females were tested for each combination (receiver vs donor: male vs. male, male vs. female, female vs. female, female vs. male) and stimulus. Each animal was taken from a pool of 20 males and 20 females kept in separate tanks (1.2 × 1.0 × 0.6 m) and used only once for each stimulus. Between trials, the Y-maze was rinsed and cleaned of debris, and clean seawater was allowed to flow through the entire maze for 15 min to remove any residual stimulus. Fourteen out of 160 individuals tested were excluded from the analysis because they remained immobile for four hours (the time limit of immobility set).

The behaviours registered in each test were (1) first choice of arm and (2) percentage of time spent in each arm (in each case when the full body entered one of the two arms). The effect of each stimulus on the first choice of arm (stimulus or control) was evaluated by a two-tailed binomial test to determine if the observed frequency was different from a random choice (50/50). The nonparametric Wilcoxon signed-ranks test was used to compare the percentage of time spent in each arm (stimulus or control).

### Spawning water tests

Two sets of experiments were designed to test the effect of spawning water on spawning. The first tested if male or female spawning water could induce spawning in conspecifics and the second tested for heterospecific responses in the closely related sympatric species *H*. *mammata*. All aquaria were filled with seawater coming from the same source and with the same physicochemical properties (22–25 °C, 35 ppt salinity). All experiments were performed at night before or at full or new moon, as sea cucumber spawning has been seen to occur most frequently at these periods^22^, and only reproductively mature sea cucumbers previously selected through a gonadal biopsy were used.

For each trial, sea cucumbers used to obtain the spawning water (the donors) were placed in a 40 × 40 × 40 cm aquarium and induced to spawn by thermal shock (TS). For the TS the donors were transferred for 10 minutes to an aquarium with 5 to 6 °C cooler water before being returned to their original aquarium. Spawning occurred within one hour for males and two hours for females. The test sea cucumbers were placed individually in a series of smaller experimental aquaria (26 × 16 × 16 cm) in the morning or the day before the experiment in order to ensure spawning was not induced by the transfer from the larger to the smaller aquarium (in which case they were not used in the experiment). The tests consisted of addition of 250 ml of female or male fresh spawning water, with or without spermatozoa, or spermatozoa in seawater, always to the same corner of the small aquarium containing either a male or a female. To test for interspecific spawning activity, 250 ml of male spawning from *H*. *arguinensis* was added to a small aquarium containing an isolated male or female *H*. *mammata*. Control aquaria received 250 ml of seawater added the same way as the test seawater. Spermatozoa were filtered from spawning water (0.7 µm pore size; Whatman, GF/F).

The results were scored (spawning or not spawning) after one or two hours for males and females, respectively. Statistical significance of percent spawning of stimuli versus the seawater control was evaluated by a Fisher’s exact test (two-tailed). Difference of time in spawning response was compared between males and females using the Mann-Whitney test.

### Tests of male water fractions

In order to characterize the active substance(s) in spawning water, tests were designed to determine whether the biological activity 1) was extractable by HLB+ universal cartridges (reverse-phase sorbent, Waters corporation, Millipore, Milford, Mass., USA), and 2) was stable. HLB+ cartridge extracts (fraction retained in cartridge and eluted in 5 ml of methanol) and filtrates (the flow-through fraction) of 1 l fresh male spawning water from which sperm and particles had been filtered, as indicated above, were obtained following the generic protocol in the manufacturer’s manual. For the spawning tests, the same experimental setup as above was used with the following stimuli: HLB cartridge extract (E), HLB cartridge filtrate (F), E and F together (E + F), fresh spawning water (FSW), spawning water aged 2 hours (2 h FSW) and spawning water aged 4 hours (4 h FSW). The solution containing extract was prepared by adding 1.25 ml of methanol extract to 250 ml of sea water (E) or 1.25 ml of methanol extract to the 250 ml filtrate (E + F). Each extract (E) and filtrate (F) was used in four tests. Two control aquaria were used, with sea water only and methanol (1.25 ml diluted in 250 ml sea water). If sea cucumbers spawned within the expected period, the test was stopped. If they did not respond, the complementary stimulus was added (E or F) or FSW. Finally, if they did not respond to FSW, a TS was provided. FSW and TS were used as positive controls to determine if unsuccessful spawning was due to the sea cucumber not being ready to spawn. Those that did not respond to any stimuli were not considered in the analysis.

Statistical significance of percent spawning of stimuli versus the seawater only control was evaluated by a Fisher’s exact test (two-tailed).

### Preliminary chemical characterization spawning water

HLB + extracts of filtered seawater taken from the same aquarium before and after spawning (males: n = 5; females: n = 2) were used for subsequent analyses by mass spectrometry. The mass spectrometer was a Bruker Esquire HCT *ultra* ion trap, equipped with an electrospray ionization source (ESI) (Agilent), operating in the negative and positive polarities. For ESI-MS^n^ studies (direct injection) the typical spray and ion optics conditions were the following: capillary voltage, 4.0 kV; nebulizer gas pressure, 30 psi; drying gas, 300 °C; drying gas flow, 6 l/min; capillary exit voltage, 208 V; skimmer voltage, 15 V. The solutions were infused into the ESI source using a syringe pump (model 781100, KDScientific, USA), at a rate of 4 μl/min. Infusion was performed using samples extracted with methanol, after washing the HLB+ cartridges with ultra-pure water. This washing step removes excess salts, which quench the formation of ions under ESI. Direct injection allowed us to obtain fragmentation spectra of order higher than 2 (MS^n^, n > 2).

The samples were also analysed by liquid chromatography (LC, Agilent Technologies 1200 Series) coupled to the above described mass spectrometer (LC-MS), under Auto-MS mode in both, positive and negative polarity. Under LC-MS operation the spray and ion optics conditions were the following: capillary voltage, 3.5 kV; drying gas (nitrogen), 330 °C at 7 l/min; nebulizer gas pressure, 35 psi; capillary exit voltage, 104 V; skimmer voltage, 32 V.

A Hamilton PRP-1 reversed phase LC column (15.0 cm length, 2.1 mm internal diameter, 5 µm average particle diameter), stabilized at 25 °C was used for chromatographic separation. The eluent system was ultra-pure water (A) and acetonitrile (B), both with 0.1% formic acid, and ethyl acetate (C). The gradient started with 52% A, 38% B and 10% C. After 5 minutes an increase of B and C up to 73% and 25%, respectively, took place over 8 minutes. The eluent was then allowed to recover to the initial conditions (52% of A, 38% of B and 10% of C) in 1 minute and then stabilize for additional 5 min before the next run. The flow was 0.35 ml/min. Full-scan mass spectra were generated in the range of 100.00–1500.00 *m/z*, both under negative and positive ESI. The data were analysed using the software Data Analysis software v 3.4 (Bruker Daltonics esquire 6.1).

Under LC-MS, a separation by LC took place before ESI-MS analysis. As salts came out from the column in the first 1-2 minutes, the flow was sent to waste. LC separation also allowed for the observation of less complex full scan spectra and for detection of compounds less prone to ionize and therefore not visible under direct injection. The AutoMS mode allowed for fragmentation (MS2) of compounds detected after LC separation. This process was done for both polarities in a single run.

Compound assignment was based on the *m/z* values, isotope distributions and fragmentation patterns. The presence of compounds possessing a phosphatidylcholine moiety was confirmed by injection of a phospholipid authentic sample, specifically compound 1,2-stearoyl phosphatidylcholine present in the standard Sigma P5394.

### Data availability

The datasets generated during and/or analysed during the current study are available from the corresponding author on request.

### Ethical approval

All applicable international, national and institutional guidelines for the care and use of animals were followed.

## Results

### Y-maze tests of attraction

For each stimulus, at least 85% of the sea cucumbers chose to move from the entry area to one of the arms. After spending some time in one of the arms, about half of the sea cucumbers went back to the entry area and re-entered the same or the other arm of the Y-maze, and eventually repeated this behaviour.

**Figure 2:**
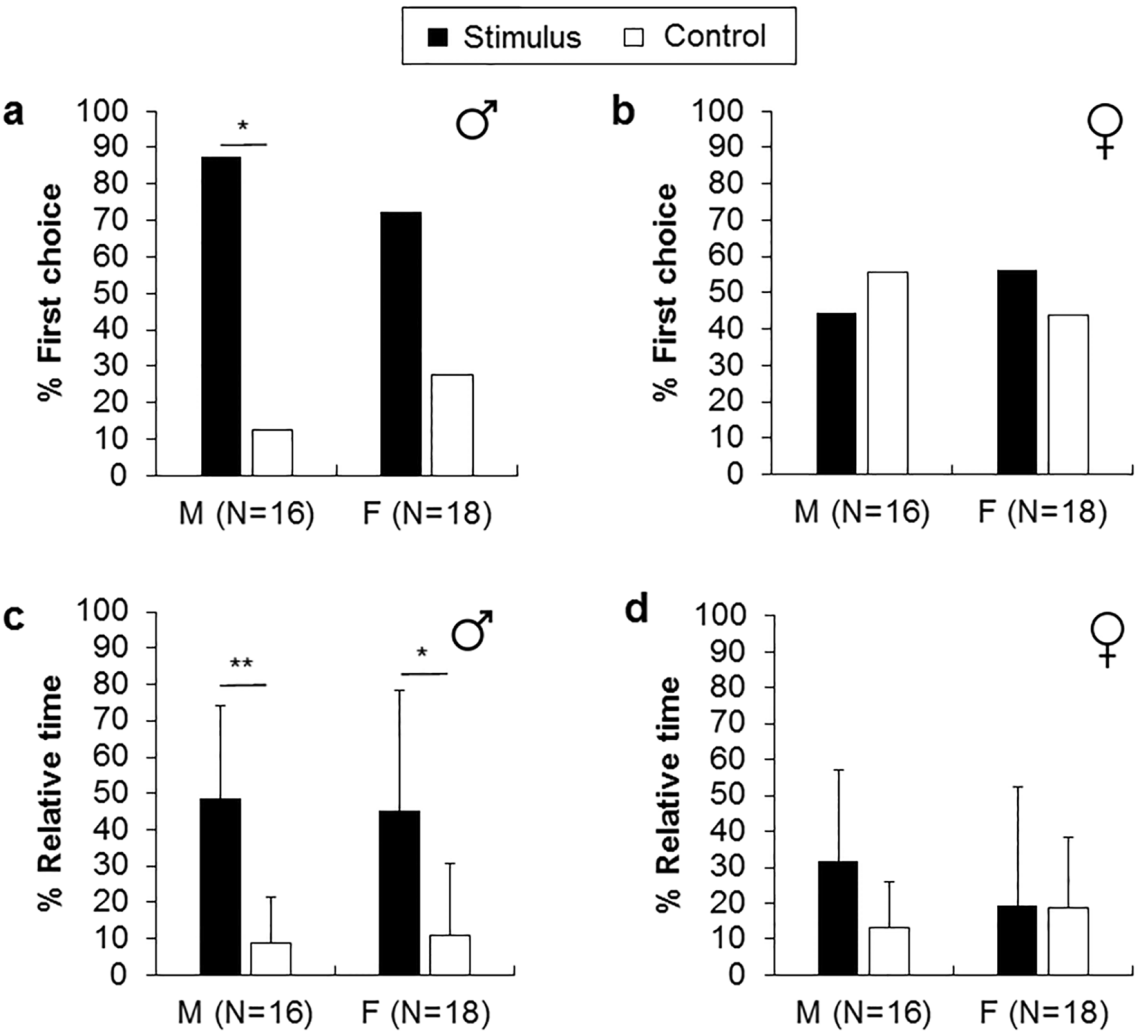
Percentage of first choice (**a**,**b**) and relative time (**c**,**d**) spent in the conditioned-conspecific water (stimulus) and in the control arm (sea water). M corresponds to male receiver and F to female receiver. Error bars are one standard deviation. *p < 0.05, **p < 0.01.

Males more often chose first the male-conditioned water arm than the control (Fig. 2a, two-tailed binomial test, p < 0.01), whereas for females there was no apparent preference (p = 0.10). Males and females showed no preference in their first decision when confronted with female-conditioned water (Fig. 2b, males: p = 0.80; females: p = 0.81). However, both males and females stayed significantly longer in the arm with the male-conditioned water (Fig. 2c, Wilcoxon signed-ranks tests, males: p < 0.01, Z = 3.21; females: p < 0.05, Z = 2.50). In contrast, males and females spent the same percentage of time in water conditioned by females and control water (Fig. 2d, males: p = 0.10, Z = 1.63; females: p = 0.90, Z = 0.13).

**Figure 3:**
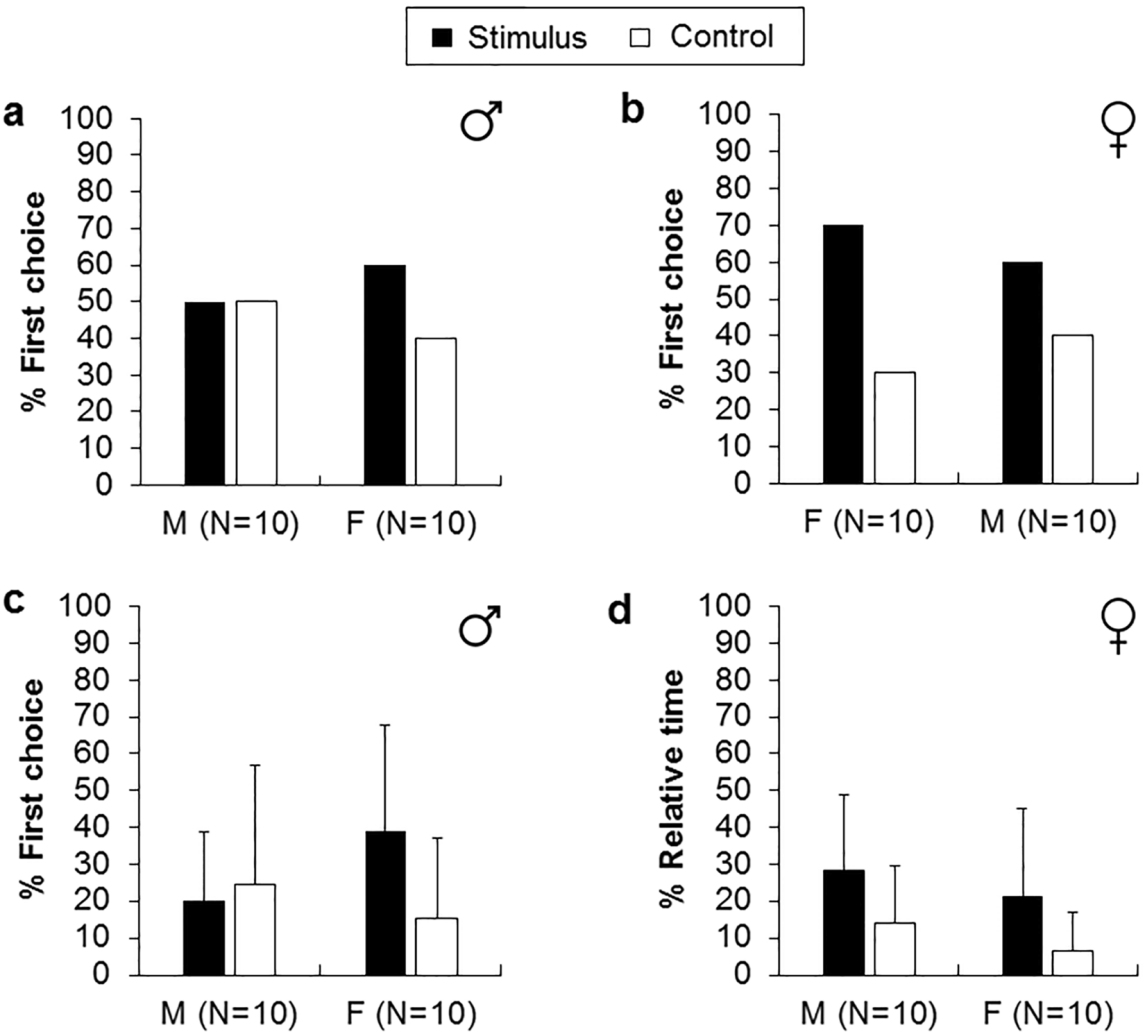
Percentage of first choice (**a**,**b**) and relative time (**c**,**d**) spent in the coelomic fluid (stimulus) and in the control arm (sea water). M corresponds to male receiver and F to female receiver. Error bars are one standard deviation.

CF from males or females did not induce first choice preference for any arm in males (Fig. 3a, two-tailed binomial tests, males-CF: p = 1.00; Fig. 3b, females-CF: p = 0.75) or females (Fig. 3a, males-CF: p = 0.75; Fig. 3b, females-CF: p = 0.34). Similarly, males and females spent similar time in the two arms when the stimulus was CF from male (Fig. 3c, Wilcoxon signed-ranks tests, males: p = 0.96, Z = 0.06; females: p = 0.15, Z = 1.43) or female (Fig. 3d, males: p = 0.39, Z = 0.87; females: p = 0.20, Z = 1.27).

**Figure 4:**
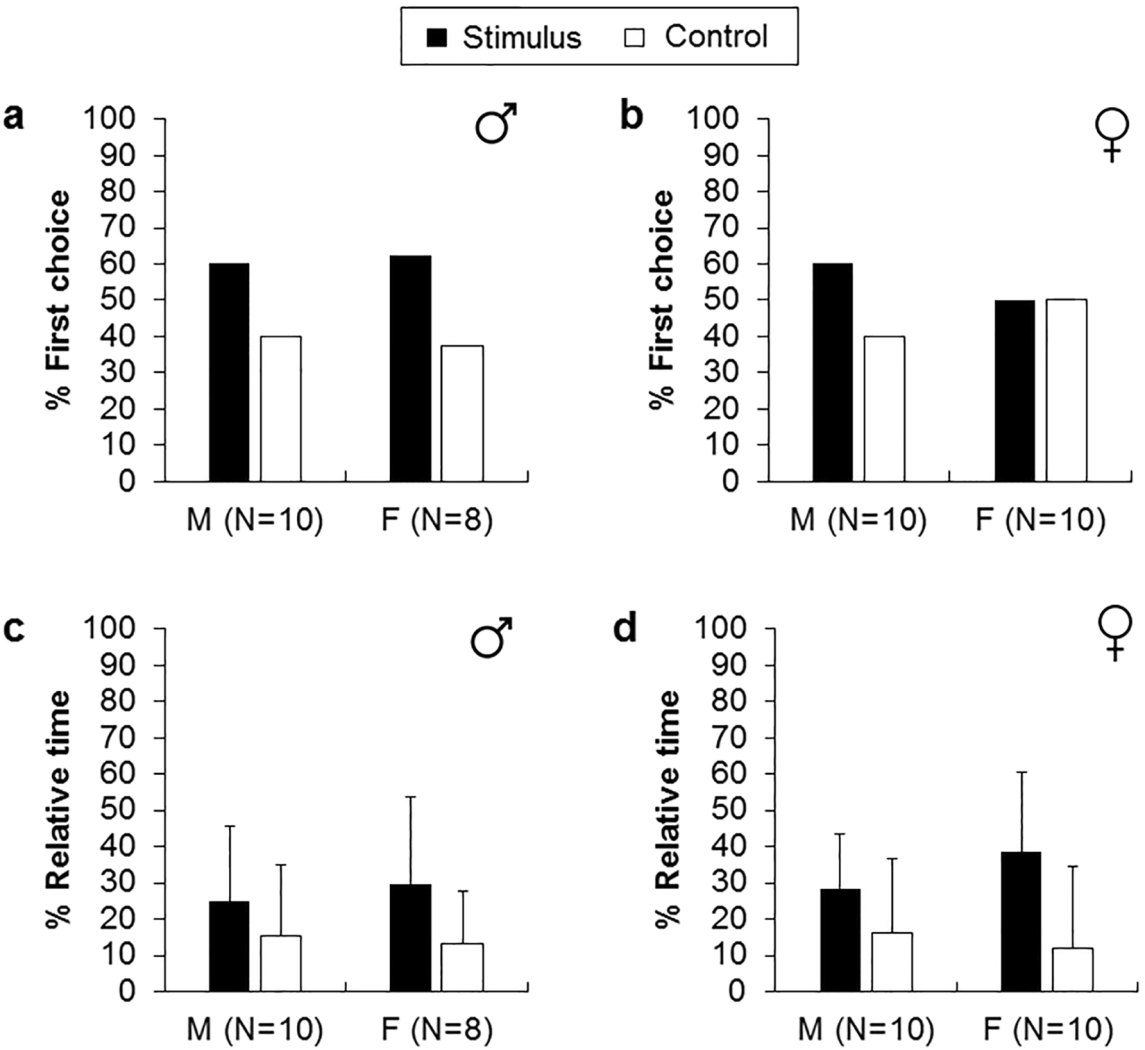
Percentage of first choice (**a**,**b**) and relative time (**c**,**d**) spent in the gonad (ovary and testis) homogenate (stimulus) and in the control arm (sea water). M corresponds to male receiver and F to female receiver. Error bars are one standard deviation.

As with the CF, ovary (O) and testis (T) homogenates failed to induce a preference for one arm either as first choice (Fig. 4a, two-tailed binomial tests, T vs. males: p = 0.75; T vs. females: p = 0.73; Fig. 4b, O vs. females: p = 1.00; O vs. males: p = 0.75) or in the time spent in one arm (Fig. 4c, Wilcoxon signed-ranks tests, T vs. males: p = 0.28, Z = 1.07; T vs. females: p = 0.14, Z = 1.48; Fig. 4d, O vs. females: p = 0.14, Z = 1.48; O vs. males: p = 0.21, Z = 1.26).

### Spawning behaviour

Thermally or chemically stimulated male and female *H*. *arguinensis* adopted a pre-spawning behaviour in which their anterior body region swayed from one side to the other, with tentacles extended outside of the oral cavity (Supplementary material 1a), while the posterior body region rested against the bottom or the side of the glass aquarium (Supplementary material 1b,c). Both males and females released gametes from a gonopore (Supplementary material 1d), located at the dorsal side of the anterior part at the opposite side of the oral cavity and was clearly visible only during spawning.

Males started to release gametes between 40 minutes and one hour after stimulation (mean: 49.70 ± 8.17 min, N = 10) and continued to slowly release a continuous flow of sperm for at least one hour (Supplementary material 2b), with some individuals still spawning after three hours. The latency of response of females was longer than that of males (Mann-Whitney *U* test, U = 16.50, N = 10, p < 0.01), varying between 50 and 100 minutes (mean: 70.30 ± 18.41 min, N = 10). However, in contrast to the continuous slow release of males, females released their gametes quickly and briefly in three to five pulsatile jets (Supplementary material 2a). Similarly, when males and females received the stimulus at the same time, males also spawned longer than females and generally continued to spawn at least until females stopped releasing gametes.

### Spawning water tests

All males and 11 of the 12 females tested spawned when male spawning water was added to their aquaria (Fisher’s exact tests, males or females, p < 0.0001), unlike with female spawning water which had no significant effect on spawning of either males or females (Table 1). If sperm was removed from the spawning water, more than three quarters of males and females still released their gametes (males or females, p < 0.001), but sperm itself had no significant effect on spawning of either sex (p > 0.05 for both). None of the males and only 1 of 16 females spawned in the control.

**Table 1:** Results of the spawning experiment. Number of males and females responding to the different tested stimulus. Fisher’s exact tests (two-tailed) were performed by comparing the percentage of individuals that responded to the different tested stimuli with the percentage of individuals responding in the control test. n.a.: not applicable (i.e. zero against zero).

Test	Spawning frequency					
*H*. *arguinensis*	Male			Female		
	Yes	No	p	Yes	No	p
Female spawning water	0	11	n.a.	3	8	0.27
Male spawning water	12	0	<0.0001	11	1	<0.0001
Male spawning water without spermatozoa	9	1	<0.0001	8	2	<0.001
Spermatozoa with sea water	2	8	0.16	1	9	1.00
Control	0	14		1	15	
***H***. ***arguinensis*** **vs**. ***H***. ***mammata***	**Male**			**Female**		
	**Yes**	**No**	**p**	**Yes**	**No**	**p**
*H*. *arguinensis* male spawning water	6	0	<0.01	5	1	<0.05
Control	0	6		0	6	

Interspecific spawning was observed between *H*. *arguinensis* and *H*. *mammata*. All males and 5/6 female *H*. *mammata* spawned when they received male spawning water from *H*. *arguinensis* in their aquarium while none of the males and females *H*. *mammata* spawned in the control (Fisher’s exact tests, males, p < 0.01; females, p < 0.05, Table 1).

### Tests of male water fractions

All sea cucumbers spawned when they received FSW and 6/7 spawned with 2 h FSW (Fisher’s exact tests, p < 0.01 in both cases; Table 2; Supplementary material 3). However, no sea cucumber released gametes with 4 h FSW (p = 1.00). When fractions E or F were added individually to the experimental tank, less than a quarter of sea cucumbers started to spawn (p > 0.05 in both cases). After the addition of the complementary stimulus, only 4/7 and 1/10 spawned with E and F, respectively (p > 0.05 in both cases). However, more than 50% of sea cucumbers spawned directly after E and F were added together (close to statistical significance, p = 0.06).

**Table 2:** Results of the stability and fraction tests experiment. Number of sea cucumbers (males and females pooled) responding to the different tested stimulus. Fisher’s exact tests (two-tailed) were performed by comparing the percentage of individuals that responded to the different tested stimuli with the percentage of individuals responding in the control test. E: HLB cartridge extract, F: HLB cartridge filtrate, E + F: E + F together, FSW: fresh spawning water, 2 h FSW: spawning water aged 2 hours, 4 h FSW: spawning water aged 4 hours, T0 and T1: times at which the first stimulus and the complementary stimulus were added respectively to the experimental aquaria.

Test	Spawning frequency		
	Yes	No	p
E (T0)	2	11	1.00
F (T0)	2	7	0.58
F (T1)	1	9	1.00
E (T1)	4	3	0.10
E + F	5	4	0.06
FSW	7	0	<0.001
2 h FSW	6	1	<0.01
4 h FSW	0	7	1.00
Control	1	9	

The sea cucumbers that did not spawn with extracts, or aged spawning water, were induced to spawn by FSW or TS. If they failed to respond to any stimuli they were not considered in the analysis (14 out of 76). Also, 20 out of 222 (9%) sea cucumbers spawned spontaneously before any stimulus was added, and were not used in experiments.

### Chemical characterization of spawning water

**Figure 5:**
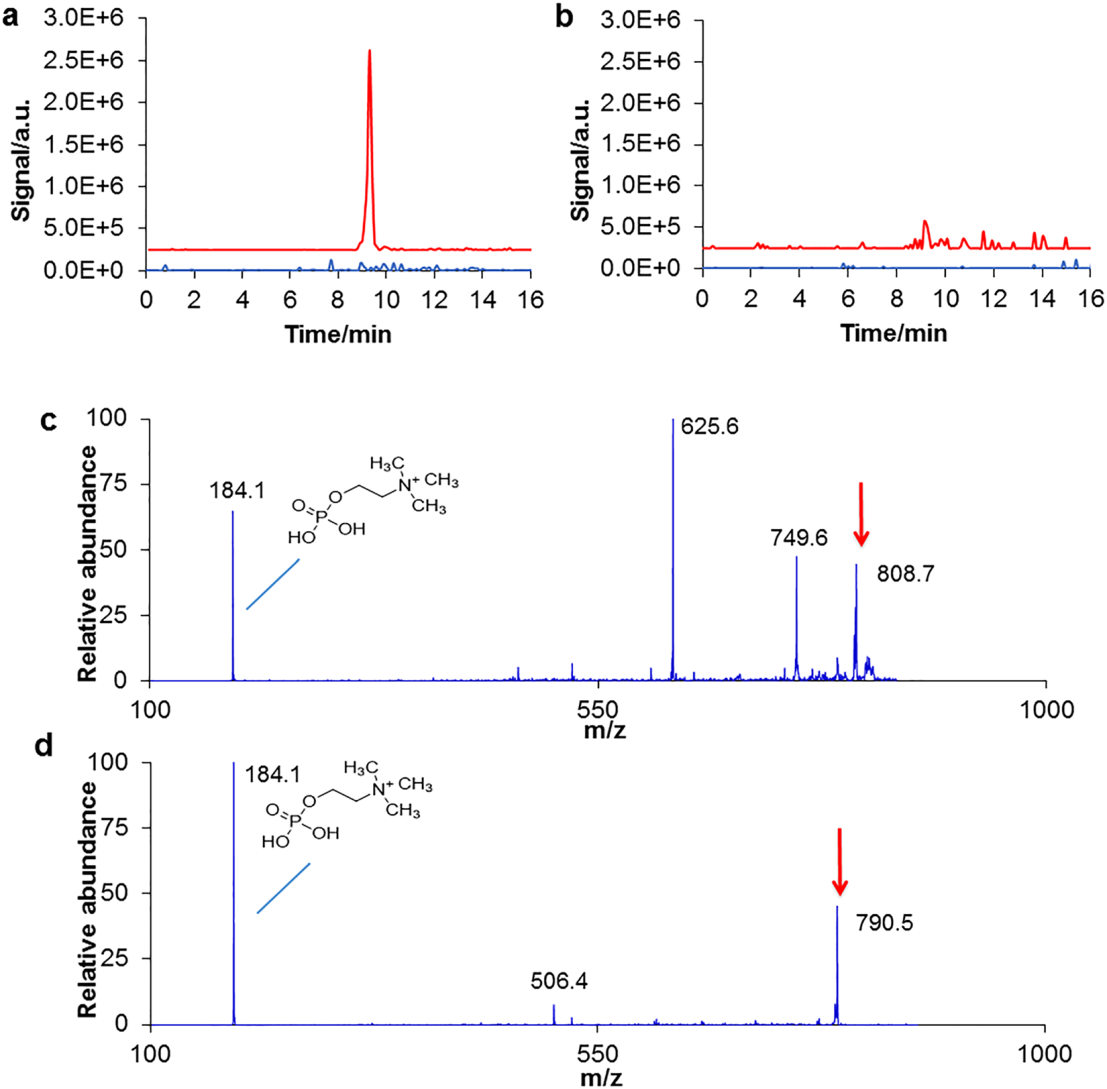
(**a**,**b**) LC-MS single ion traces of methanol extracts at *m/z* 808.7 under positive polarity. (**a)** Male before (blue) and after (red) spawning; (**b**) female before (blue) and after (red) spawning. (**c**,**d**) Fragmentation spectra. (**c**) MS2(808.7); (**d**) MS2(790.5), the standard 1,2-stearoyl phosphatidylcholine. The inserted structures were assigned to ion with *m/z* 184.1. The arrows indicate the fragmented peaks.

Full scan LC-MS profiles of water extracted before and after spawning of males and females were clearly different under both ionization polarities (Supplementary material 4). Since only male spawning water was active, only these LC-MS profiles were analysed further. Major differences between samples obtained before and after spawning could be seen between 8 and 11 min. Among those compounds detected by MS, the most intense was found at *m/z* 808.7 (positive polarity) in the water after spawning, and was absent before spawning (Fig. 5a). A much smaller peak of this compound was also seen in female spawning water (Fig. 5b). Under positive polarity, Auto MS gave a fragmentation spectrum showing a major signal at *m/z* 184.1 (Fig. 5c). This result was confirmed by direct injection of the sample into the mass spectrometer (ESI-MS). The *m/z* value and the daughter ion at *m/z* 184.1 under positive polarity are common in phospholipids possessing a phosphatidylcholine moiety^49^. To evaluate the presence of a phosphatidylcholine moiety, the phosphatidylcholine phospholipid standard 1,2-stearoyl phosphatidylcholine was selected and studied by ESI-MS^n^. This compound is readily seen at *m/z* 790.5 under positive polarity as it contains a net positive charge. The fragmentation led to a major signal at *m/z* 184.1 (Fig. 5d), which is the same daughter ion observed for the fragmentation of *m/z* 808.7. This result suggests that the unknown compound might possess a phosphatidylcholine moiety.

**Figure 6:**
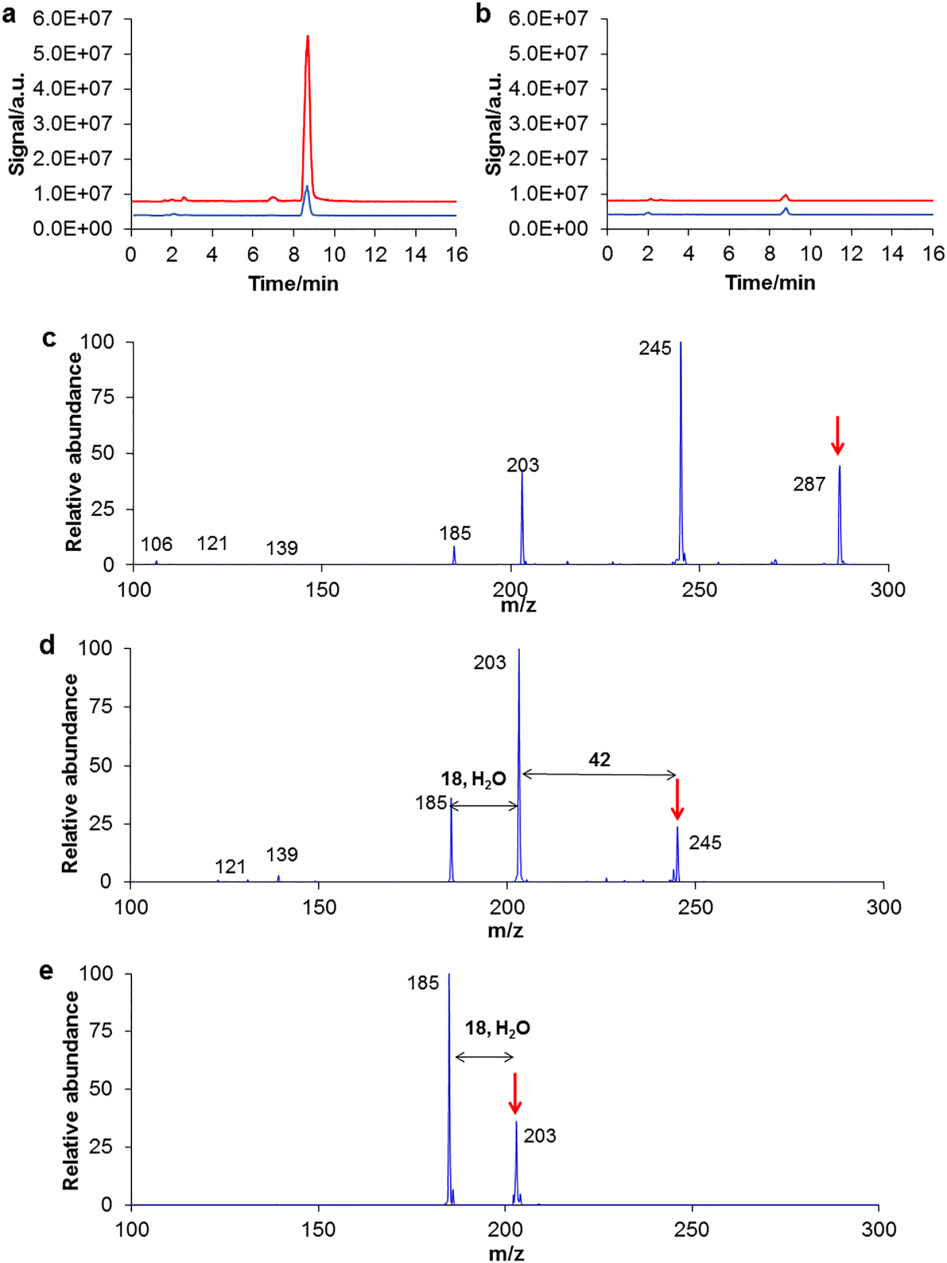
(**a**,**b**) LC-MS single ion traces of methanol extracts at *m/z* 287 under positive polarity. (**a**) Male before (blue) and after (red) spawning; (**b**) female before (blue) and after (red) spawning. (**c**–**e**) Fragmentation spectra of *m/z* 287. (**c**) MS2(287); (**d**) MS3(287→245); (**e**) MS4(287→245→203). The arrows indicate the fragmented peaks.

Another signal detected by LC-MS that could be associated with male spawning was seen at *m/z* 287 under positive polarity (Fig. 6a). This signal was seen in male water prior to spawning but increased greatly after spawning. A much smaller peak was seen in female water which did not increase after spawning (Fig. 6b). The fragmentation spectra are shown in Fig. 6c–e.

The signal intensity of the two compounds increased with time with a maximal intensity after 30 min and 90 min after the beginning of the spawning process for the *m/z* 808.7 and *m/z* 287, respectively. Their signal intensity then decreased progressively, even though the sea cucumber continued to release sperm. This is consistent with the reduction of spawning activity seen in the bioassay (Supplementary material 5).

## Discussion

The present study demonstrates that chemical cues produced by male sea cucumbers attract conspecifics and trigger spawning in both sexes, indicating an important role of the chemosensory system in the coordination of aggregation and spawning behaviours.

Pre-spawning males and females spent more time in the arm of the Y-maze with male-conditioned water. This indicates that males release (a) chemical(s) to the water that are attractive to both sexes. The coelomic fluid and gonad homogenates (ovary and testis) did not attract either sex and are therefore unlikely sources of aggregation odorants. This contrasts with the avoidance reaction of sea urchins, when confronted with conspecific coelomic fluid or gonad extract^50,51^. However, in the present study, the gonad extracts and coelomic fluid were frozen before tested and active compounds in these homogenates could have degraded; it is pertinent to add that these experiments with gonad extracts and coelomic fluid were carried out before the spawning experiments wherein the labile nature of the spawning pheromone became apparent. Another possible source of odorants is the mucus of mature individuals which, in *Cucumaria frondosa*, has been shown to accelerate gonadal development of less mature individuals^52^. However, we have no indication that more mucus is released during spawning than at any other times or that it is a source of pheromones in the species we tested.

That only the males produce/release the aggregation pheromone could be a strategy to draw sea cucumbers to the same place to spawn, while limiting sperm dispersion through male-male groups and maximizing fertilization success through male-female groups^6,15^. Specific male aggregative behaviours have also been reported in brittle stars^53^ and sex recognition by mechano-reception in male starfishes^54^. Recently, sedentary starfish *A*. *planci* were seen to be attracted to water-borne chemical plumes released from aggregating individuals^55^, which supports the presence of aggregation pheromones in echinoderms. As described briefly above, *H*. *arguinensis* performs a ‘nuptial’ sequence before spawning, which culminates in gamete release and a sperm mass that slowly disperses. Whether, in the wild, this happens in pairs or in a promiscuous mating mode is not known. Further investigations are needed to better understand the triggers and benefits of sea cucumber breeding aggregations and to determine if male attraction is also present outside the pre-spawning period.

Male spawning water, with or without spermatozoa, induced spawning in males and females, whereas female spawning water had no effect. This suggests that males release (a) chemical(s) during spawning which stimulate(s) both sexes to release gametes. To our knowledge, it is the first time this has been shown in sea cucumbers, and is consistent with what has been observed in sea urchins^31^ and starfish^29^, although in those studies spermatozoa were not removed from water.

Consistent with previous studies of marine broadcast spawners, males were quicker to release their gametes than females, a feature that has been suggested to be favoured by sexual selection when males are competing to fertilize the ova, so as to enable fertilization of more eggs over larger areas^25,56,57^. In the present study, males also spawned longer than females; i.e. they continued to spawn at least until females stopped and beyond. This behaviour has been reported in other Holothuroidea^23,28^ and in other marine broadcast spawners such as Ophiuroidea^58^, Echinoidea^27^, Polychaeta^11,59^ and Appendicularia^60^. Releasing sperm more slowly than eggs was shown to be a good strategy to avoid sperm attaching uselessly to fertilized eggs, since the permanent block preventing subsequent sperm attachment to the eggs takes longer to form than the first block preventing polyspermy^61,62^.

Interestingly, as with *H*. *arguinensis*, and with similar efficacy, male and female *H*. *mammata* released their gametes in response to *H*. *arguinensis* male spawning water. This suggests that the two species use the same or similar chemical signals. Heterospecific spawning inducing activity has been documented in other Holothurians and in other invertebrates^10,23,63–65^ and has been suggested to result from the coevolution of pheromones in response to reduced predation risks through predator swamping, since isolated spawn clumps from one species are more likely to be caught when they are isolated than when they are grouped with those from another species^66^. This observation raises the possibility of inter-specific hybridization between the two species, a phenomenon which has been documented among other sea cucumber species^67,68^, although species-specific circadian patterns and spawning behaviours minimize this possibility^69^.

Spawning was rarely induced when male spawning water was 4 hr old, indicating degradation or evaporation of spawning substance(s). Similarly, separate addition of the eluate or filtrate had no activity, which could be at least partly restored if the complementary fraction was added subsequently. This suggests that the spawning pheromone consists of more than one compound. However, solid-phase extraction of water took between one and three hours; and some loss of activity of the extracts could therefore be explained by degradation of the active compound(s) during this time.

In this study, two compounds were found in male spawning water under positive polarity, at *m/z* 808.1 and *m/z* 287, while they were absent or present at much lower concentrations in female water and before male spawning. Both showed a decrease within two hours, consistent with the loss of biological activity of male spawning water. They are thus good candidates to be involved in sea cucumber spawning. The fragmentation of compound with *m/z* 808.1 suggests the presence of a phosphatidylcholine moiety. Phosphatidylcholines are a major component of the plasma membrane and are known to be involved in sperm motility^70^, the acrosomal reaction^71^ and in the maintenance of sperm membrane integrity^72^. Moreover, phosphatidylcholines have been characterized as key substances governing group recognition in catfish^73^ and as phagostimulants in the nuptial secretion of a species of cockroach^74^. However, to our knowledge, they have never been associated with spawning activity in any animal taxon.

Saponins are among the most important and abundant secondary metabolites of sea cucumbers, and have been reported to be involved in the chemical communication of marine organisms^75,76^. Although we cannot exclude the involvement of saponins in the spawning process, none of the detected compounds show mass spectral properties consistent with the reported for sea cucumber saponins. While the compound with *m/z* 287 is too small to be a saponin and was not detected under negative polarity, the fragmentation of compound with *m/z* 808.1 does not release water upon fragmentation (Fig. 5c), a typical neutral loss of compounds possessing sugar moieties, including saponins^76^.

Pheromones are highly diverse across different animal taxa, and are composed either of a mixture of different chemical compounds or a single compound^77^. Some fish species use steroids and/or prostaglandins as sex pheromones^78^ while others use amino acids^79^ or bile acids^80^. In marine invertebrates, for example, peptide pheromones have been identified in the sea-slug *Aplysia* and nereid worms^81–83^ and nucleotide pheromones in crustaceans^84^.

The chemical identification of pheromones is challenging due to the relatively small amount of pheromone released, to the large variety of substances usually present in natural waters, and to their possible lability^85^. Here, the compounds correlated with spawning need to be isolated, purified and submitted to further analysis, namely nuclear magnetic resonance (NMR) and high-resolution mass spectrometry to assign a final structure. The identified compounds would need then to be tested on the animal to confirm their biological activity. It would be also of great interest to know the source of the pheromones and their regulation.

Reproductive success in sea cucumbers results is likely dependent upon a combination of chemical cues and one or more exogenous factors^31,86^. This study provides evidence that sea cucumbers use pheromones for aggregation and spawning, and their partial characterization could have important ecological and practical applications in the management of sea cucumbers in aquaculture, and as an attractant in the control of invasive species, *viz a viz* their use in the control of pest insects^87^ and invasive sea lamprey^88^.

## Electronic supplementary material

Supplementary materials

## References

[1] Giese, A. C. & Kanatani, H. Maturation and spawning. In *Reproduction of marine invertebrates*, *Vol*. *IX* (eds Giese, A. C., Pearse, J. S. & Pearse, V. B.) 251–329 (Blackwell Scientific/Boxwood Press, 1987).

[2] Wray, G. A. Evolution of larvae and developmental modes. In *Ecology of marine invertebrate larvae* (ed L. McEdward) 412–448 (CRC, 1995).

[3] Denny MW, Shibata MF (1989). Consequences of surf-zone turbulence for settlement and external fertilization. Am. Nat..

[4] Pennington JT (1985). The ecology of fertilization of echinoid eggs: the consequences of sperm dilution, adult aggregation, and synchronous spawning. Biol. Bull..

[5] Yund PO (2000). How severe is sperm limitation in natural populations of marine free-spawners?. Trends Ecol. Evol..

[6] Levitan DR, Petersen C (1995). Sperm limitation in the sea. Trends Ecol. Evol..

[7] Babcock R (1995). Synchronous multispecific spawning on coral reefs: potential for hybridization and roles of gamete recognition. Reprod. Fert. Develop..

[8] Giese, A. C., Pearse, J. S. & Pearse, V. *Reproduction of marine invertebrates*, *Vol*. *6: Echinoderms and Lophophorates*. (Boxwood Press, 1991).

[9] Hamel J-F, Mercier A (1996). Evidence of chemical communication during the gametogenesis of holothurids. Ecology.

[10] Watson GJ, Bentley MG, Gaudron SM, Hardege JD (2003). The role of chemical signals in the spawning induction of polychaete worms and other marine invertebrates. J. Exp. Mar. Biol. Ecol..

[11] Hardege JD, Bentley MG (1997). Spawning synchrony in *Arenicola marina*: evidence for sex pheromonal control. Proc. R. Soc. Lond. B.

[12] Soong K, Chang D, Chao SM (2005). Presence of spawn-inducing pheromones in two brittle stars (Echinodermata: Ophiuroidea). Mar. Ecol. Prog. Ser..

[13] Painter, S. D., Clough, B., Garden, R. W., Sweedler, J. V. & Nagle, G. T. Characterization of *Aplysia* attractin, the first water-borne peptide pheromone in invertebrates. *Biol*. *Bull*. **194**, 10.2307/1543042 (1998). 10.2307/1543042 9604313

[14] Bamber S, Naylor E (1996). Chemical communication and behavioural interaction between sexually mature male and female shore crabs (*Carcinus maenas*). J. Mar. Biol. Assoc. U.K..

[15] Levitan DR, Sewell MA, Chia F-S (1992). How distribution and abundance influence fertilization success in the sea urchin *Strongylocentotus franciscanus*. Ecology.

[16] Levitan DR (1991). Influence of body size and population density on fertilization success and reproductive output in a free-spawning invertebrate. Biol. Bull..

[17] Tominaga H, Nakamura S, Komatsu M (2004). Reproduction and development of the conspicuously dimorphic brittle star *Ophiodaphne formata* (Ophiuroidea). Biol. Bull..

[18] Mercier A, Hamel J-F (2008). Depth-related shift in life history strategies of a brooding and broadcasting deep-sea asteroid. Mar. Biol..

[19] Leite-Castro LV (2016). Reproductive biology of the sea cucumber *Holothuria grisea* in Brazil: importance of social and environmental factors in breeding coordination. Mar. Biol..

[20] Young CM, Tyler PA, Cameron JL, Rumrill SG (1992). Seasonal breeding aggregations in low-density populations of the bathyal echinoid *Stylocidaris lineata*. Mar. Biol..

[21] Beach, D. H., Hanscomb, N. J. & Ormond, B. F. G. Spawning pheromone in crown-of-thorns starfish. *Nature* **254**, 10.1038/254135a0 (1975). 10.1038/254135a0 1117997

[22] Mercier A, Hamel J-F (2009). Endogenous and exogenous control of gametogenesis and spawning in Echinoderms. Adv. Mar. Biol..

[23] McEuen FS (1988). Spawning behaviors of northeast Pacific sea cucumbers (Holothuroidea: Echinodermata). Mar. Biol..

[24] Miller RL (1989). Evidence for the presence of sexual pheromones in free-spawning starfish. J. Exp. Mar. Biol. Ecol..

[25] Thorson G (1950). Reproductive and larval ecology of marine bottom invertebrates. Biol. Rev..

[26] Himmelman JH, Dumont CP, Gaymer CF, Vallières C, Drolet D (2008). Spawning synchrony and aggregative behaviour of cold-water echinoderms during multi-species mass spawnings. Mar. Ecol. Prog. Ser..

[27] Levitan DR (2002). Density-dependent selection on gamete traits in three congeneric sea urchins. Ecology.

[28] Hamel JF, Mercier A (1995). Prespawning behavior, spawning, and development of the brooding starfish *Leptasterias polaris*. Biol. Bull..

[29] Caballes CF, Pratchett MS (2017). Environmental and biological cues for spawning in the crown-of-thorns starfish. PloS ONE.

[30] Starr M, Himmelman JH, Therriault JC (1990). Direct coupling of marine invertebrate spawning with phytoplankton blooms. Science.

[31] Reuter KE, Levitan DR (2010). Influence of sperm and phytoplankton on spawning in the echinoid *Lytechinus variegatus*. Biol. Bull..

[32] Kato S (2009). Neuronal peptides induce oocyte maturation and gamete spawning of sea cucumber. Apostichopus japonicus. Dev. Biol..

[33] Chaet AB, McConnaughy RA (1959). Physiologic activity of nerve extracts. Biol. Bull..

[34] Kanatani, H. Maturation-inducing substance in starfishes. In *International review of cytology* Vol. 35 (eds Danielli, J. F., Bourne, G. H. & Jeon, K. W.) 253–298 (Academic Press, 1973). 10.1016/s0074-7696(08)60356-3 4205954

[35] Mercier A, Hamel J-F (2002). Perivisceral coelomic fluid as a mediator of spawning induction in tropical holothurians. Invertebr. Reprod. Dev..

[36] Anderson SC, Flemming JM, Watson R, Lotze HK (2011). Serial exploitation of global sea cucumber fisheries. Fish. Fish..

[37] González-Wangüemert M, Valente S, Henriques F, Domínguez-Godino JA, Serrão EA (2016). Setting preliminary biometric baselines for new target sea cucumbers species of the NE Atlantic and Mediterranean fisheries. Fish. Res..

[38] Domínguez-Godino JA, Slater MJ, Hannon C, González-Wangüermert M (2015). A new species for sea cucumber ranching and aquaculture: breeding and rearing of *Holothuria arguinensis*. Aquaculture.

[39] Marquet N, Conand C, Power DM, Canário AVM, González-Wangüemert M (2017). Sea cucumbers, *Holothuria arguinensis* and *H*. *mammata*, from the southern Iberian Peninsula: variation in reproductive activity between populations from different habitats. Fish. Res.

[40] Hamel J-F, Conand C, Pawson DL, Mercier A (2001). Biology of the sea cucumber *Holothuria scabra* (Holothuroidea: Echinodermata) and its exploitation as beche-de-mer. Adv. Mar. Biol..

[41] Battaglene SC (1999). Culture of tropical sea cucumbers for stock restoration and enhancement. Naga.

[42] James DB (1994). See production in sea cucumbers. Aquac. Int..

[43] Yanagisawa, T. Aspects of the biology and culture of the sea cucumber. In *Tropical Mariculture* (ed. De Silva, S. S.) 292–308 (Academic Press, 1998).

[44] Léonet A, Rasolofonirina R, Wattiez R, Jangoux M, Eeckhaut I (2009). A new method to induce oocyte maturation in holothuroids (Echinodermata). Invertebr. Reprod. Dev..

[45] Witzgall P, Kirsch P, Cork A (2010). Sex pheromones and their impact on pest management. J. Chem. Ecol..

[46] Antoniadou C, Vafidis D (2009). Updated distribution of the holothuroid *Synaptula reciprocans* (Forskal, 1775) in the Mediterranean: does it follow shallow-water circulation patterns?. Aquat. Invasions.

[47] Galil BS (2009). Taking stock: inventory of alien species in the Mediterranean sea. Biol. Invasions.

[48] Borrero-Pérez GH, Gómez-Zurita J, González-Wangüemert M, Marcos C, Pérez-Ruzafa A (2010). Molecular systematics of the genus Holothuria in the Mediterranean and Northeastern Atlantic and a molecular clock for the diversification of the Holothuriidae (Echinodermata: Holothuroidea). Mol. Phylogenet. Evol..

[49] Hsu FF, Turk J (2009). Electrospray ionization with low-energy collisionally activated dissociation tandem mass spectrometry of glycerophospholipids: mechanisms of fragmentation and structural characterization. J. Chromatogr. B Analyt. Technol. Biomed. Life Sci..

[50] Campbell AC, Coppard S, D’Abreo C, Tudor-Thomas R (2001). Escape and aggregation responses of three echinoderms to conspecific stimuli. Biol. Bull..

[51] Mann KH, Wright JLC, Welsford BE, Hatfield E (1984). Responses of the sea urchin *Strongylocentrotus droebachiensis* (O.F. Müller) to water-borne stimuli from potential predators and potential food algae. J. Exp. Mar. Biol. Ecol..

[52] Hamel J-F, Mercier A (1999). Mucus as a mediator of gametogenic synchrony in the sea cucumber *Cucumaria frondosa* (Holothuroidea: Echinodermata). J. Mar. Biol. Ass. U.K..

[53] Hagman DK, Vize PD (2003). Mass spawning by two brittle star species,*Ophioderma rubicundum* and *O*. *Squamosissimum* (Echinodermata: Ophiuroidea), at the Flower Garden Banks, Gulf of Mexico. B. Mar. Sci..

[54] Run J-Q, Chen C-P, Chang K-H, Chia F-S (1988). Mating behaviour and reproductive cycle of *Archaster typicus* (Echinodermata: Asteroidea). Mar. Biol..

[55] Hall MR (2017). The crown-of-thorns starfish genome as a guide for biocontrol of this coral reef pest. Nature.

[56] Levitan, D. R. Sperm limitation, gamete competition, and sexual selection in external fertilizers. In *Sperm competition and sexual selection* (eds Birkhead, T. R. & Møller, A. P.) 173–215 (Academic Press, 1998).

[57] Levitan DR (2005). Sex-specific spawning behavior and its consequences in an external fertilizer. Am. Nat..

[58] Selvakumaraswamy P, Byrne M (2000). Reproduction, spawning, and development of 5 ophiuroids from Australia and New Zealand. Invert. Biol..

[59] Hardege JD, Bartels-Hardege HD, Hardege JD (1995). Spawning behaviour and development of *Perinereis nuntia* var. *brevicirrus* (Annelida: Polychaeta). Invertebr. Biol..

[60] Miller RL (2005). Gamete interactions and fertilization behavior in the larvacean. Oikopleura dioica. Invertebr. Reprod. Dev..

[61] Gould MC, Stephano JL (2003). Polyspermy prevention in marine invertebrates. Microsc. Res. Tech..

[62] Marshall DJ, Bolton TF (2007). Sperm release strategies in marine broadcast spawners: the costs of releasing sperm quickly. J. Exp. Biol..

[63] Babcock R, Mundy C, Keesing J, Oliver J (1992). Predictable and unpredictable spawning events: *in situ* behavioural data from free-spawning coral reef invertebrates. Invertebr. Reprod. Dev..

[64] Sewell MA, Levitan DR (1992). Fertilization success during a natural spawning of the Dendrochirote sea cucumber *Cucumaria miniata*. Bull. Mar. Sci..

[65] Van Veghel MLJ (1993). Multiple species spawning on Curaçao Reefs. Bull. Mar. Sci..

[66] Harrison PL (1984). Mass spawning in tropical reef corals. Science.

[67] Engstrom NA (1980). Reproductive cycles of *Holothuria (Halodeima) floridana*, *H*. *(H*.*) mexicana* and their hybrids (Echinodermata: Holothuroidea) in southern Florida, USA. Invertebr. Reprod. Dev..

[68] Uthicke S, Purcell S, Blockmans B (2005). Natural hybridization does not dissolve species boundaries in commercially important sea cucumbers. Biol. J. Linn. Soc..

[69] Mercier A, Hamel J-F (2010). Synchronized breeding events in sympatric marine invertebrates: role of behavior and fine temporal windows in maintaining reproductive isolation. Behav. Ecol. Sociobiol..

[70] Mita M, Harumi T, Suzuki N, Ueta N (1991). Localization and characterization of phosphatidylcholine in sea urchin spermatozoa. J. Biochem..

[71] Cross NL (1994). Phosphatidylcholine enhances the acrosomal responsiveness of human sperm. J. Androl..

[72] Long JA, Conn TL (2012). Use of phosphatidylcholine to improve the function of turkey semen stored at 4 degrees C for 24 hours. Poult. Sci..

[73] Matsumura K, Matsunaga S, Fusetani N (2007). Phosphatidylcholine profile-mediated group recognition in catfish. J. Exp. Biol..

[74] Kugimiya S, Nishida R, Kuwahara Y, Sakuma M (2002). Phospholipid composition and pheromonal activity of nuptial secretion of the male German cockroach. Blattella germanica. Entomol. Exp. Appl..

[75] Caulier G, Flammang P, Gerbaux P, Eeckhaut I (2013). When a repellent becomes an attractant: harmful saponins are kairomones attracting the symbiotic Harlequin crab. Sci. Rep..

[76] Bahrami Y, Zhang W, Franco C (2014). Discovery of novel saponins from the viscera of the sea cucumber *Holothuria lessoni*. Mar. Drugs.

[77] Wyatt TD (2010). Pheromones and signature mixtures: defining species-wide signals and variable cues for identity in both invertebrates and vertebrates. J. Comp. Physiol. A.

[78] Stacey, N. Hormonally derived pheromones in teleost fishes. In *Fish Pheromones and Related Cues* 33–88 (John Wiley & Sons, Inc, 2014).

[79] Yambe H (2006). L-Kynurenine, an amino acid identified as a sex pheromone in the urine of ovulated female masu salmon. Proc. Natl. Acad. Sci. USA.

[80] Li W (2002). Bile acid secreted by male sea lamprey that acts as a sex pheromone. Science.

[81] Cummins SF, Schein CH, Xu Y, Braun W, Nagle GT (2005). Molluscan attractins, a family of water-borne protein pheromones with interspecific attractiveness. Peptides.

[82] Hardege JD, Bartels-Hardege H, Muller CT, Beckmann M (2004). Peptide pheromones in female *Nereis succinea*. Peptides.

[83] Zeeck E, Hardege J, Bartels-Hardege H, Wesselmann G (1988). Sex pheromone in a marine polychaete: determination of the chemical structure. J. Exp. Zool..

[84] Hardege JD (2011). Identification of a female sex pheromone in *Carcinus maenas*. Mar. Ecol. Prog. Ser..

[85] Shimizu Y (1985). Bioactive marine natural products, with emphasis on handling of water-soluble compounds. J Nat Prod.

[86] McCarthy DA, Young CM (2002). Gametogenesis and reproductive behavior in the echinoid *Lytechinus variegatus*. Mar. Ecol. Prog. Ser..

[87] Cook SM, Khan ZR, Pickett JA (2007). The use of push-pull strategies in integrated pest management. Annu. Rev. Entomol..

[88] Siefkes MJ (2017). Use of physiological knowledge to control the invasive sea lamprey (*Petromyzon marinus*) in the Laurentian Great Lakes. Conserv. Physiol..

