# Supplementary material for: Chemicals released by male sea cucumber mediate aggregation and spawning behaviours
Source: Sci Rep. 2018 Jan 10;8:239. doi: 10.1038/s41598-017-18655-6 (PMC5762768; doi:10.1038/s41598-017-18655-6)
Supplement: Supplementary file 1 — Supplementary materials [file 41598_2017_18655_MOESM1_ESM.pdf]

Chemicals released by male sea cucumber mediate aggregation and spawning behaviours

Nathalie Marquet, Peter C. Hubbard, José P. da Silva, João Afonso, Adelino V.M. Canário

CCMAR Centre of Marine Sciences, Universidade do Algarve, Campus de Gambelas, 8005-139 Faro, Portugal

[#]Corresponding author: N. Marquet

e-mail: nmarquet@gmail.com

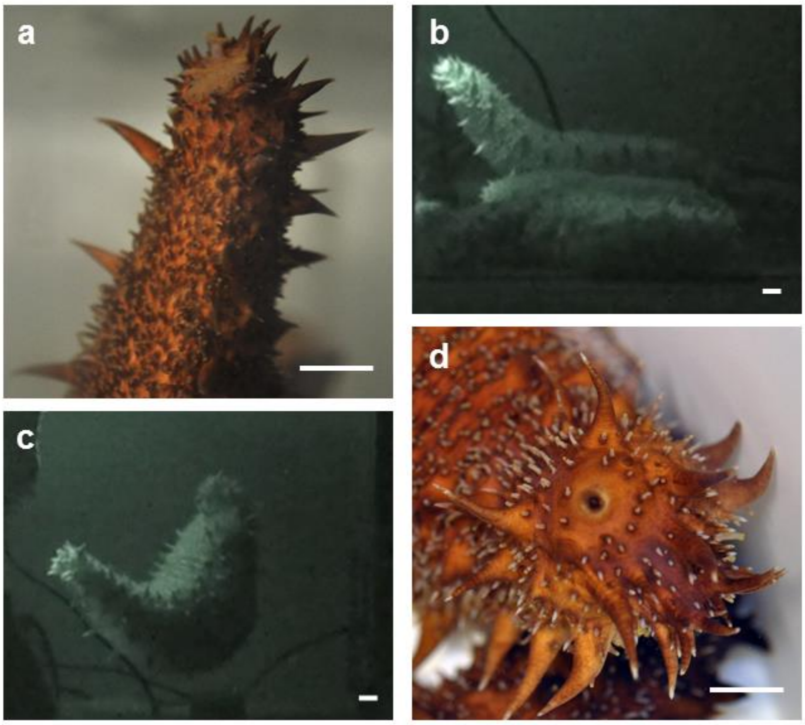

Supplementary material 1. Spawning behaviour in *H. arguinensis* showing (**a**) the tentacles outside of the oral cavity, (**b, c**) the posture of the complete body during spawning in the dark and (**d**) the gonopore from where the gametes are released. Scale bars: 1 cm.

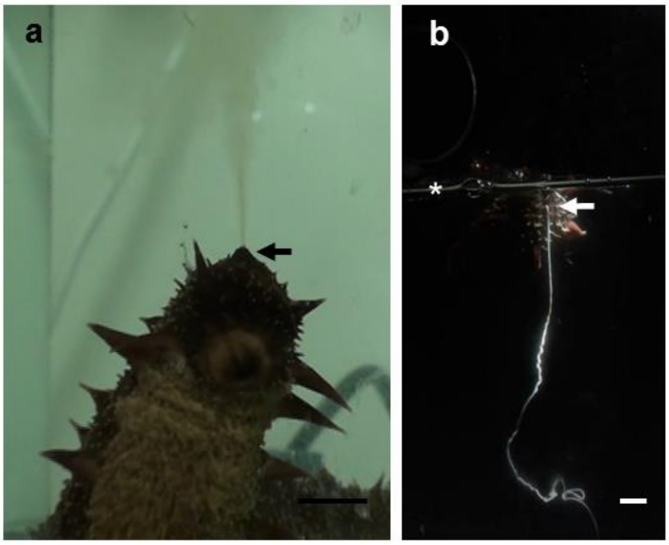

Supplementary material 2. Gamete release by a female (**a**) and a male (**b**) in *H. arguinensis*. Arrows indicates the gonopore from where gametes are released. The female is on the bottom of the aquarium while the male has his posterior part attached to one side glass of the aquarium and releases the sperm from the water surface (asterisk) to the bottom of the aquarium. Scale bars: 1 cm.

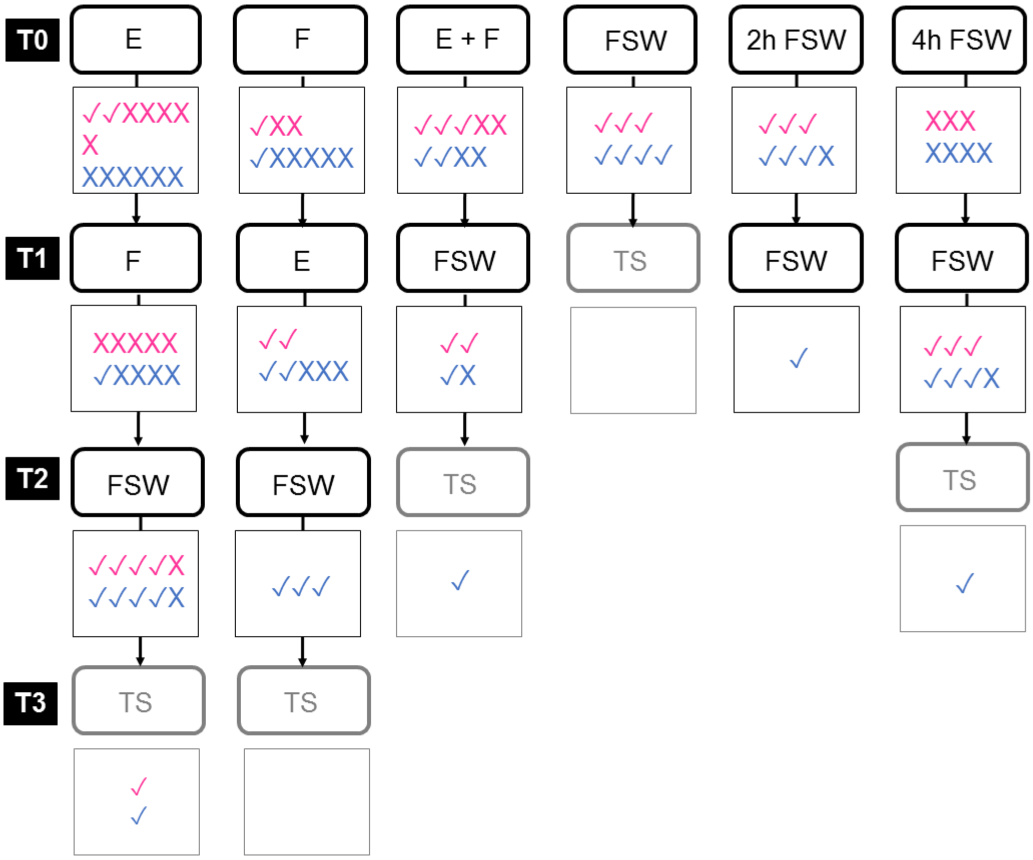

Supplementary material 3. Experimental design and results obtained in the extract vs. filtrate experiment. E: extract, F: filtrate, FSW: fresh spawning water, 2h FSW: spawning from 2hours, 4h FSW: spawning water from 4 hours, TS: thermal shock, ✓: spawning, X: no spawning, in pink: female, in blue: male.

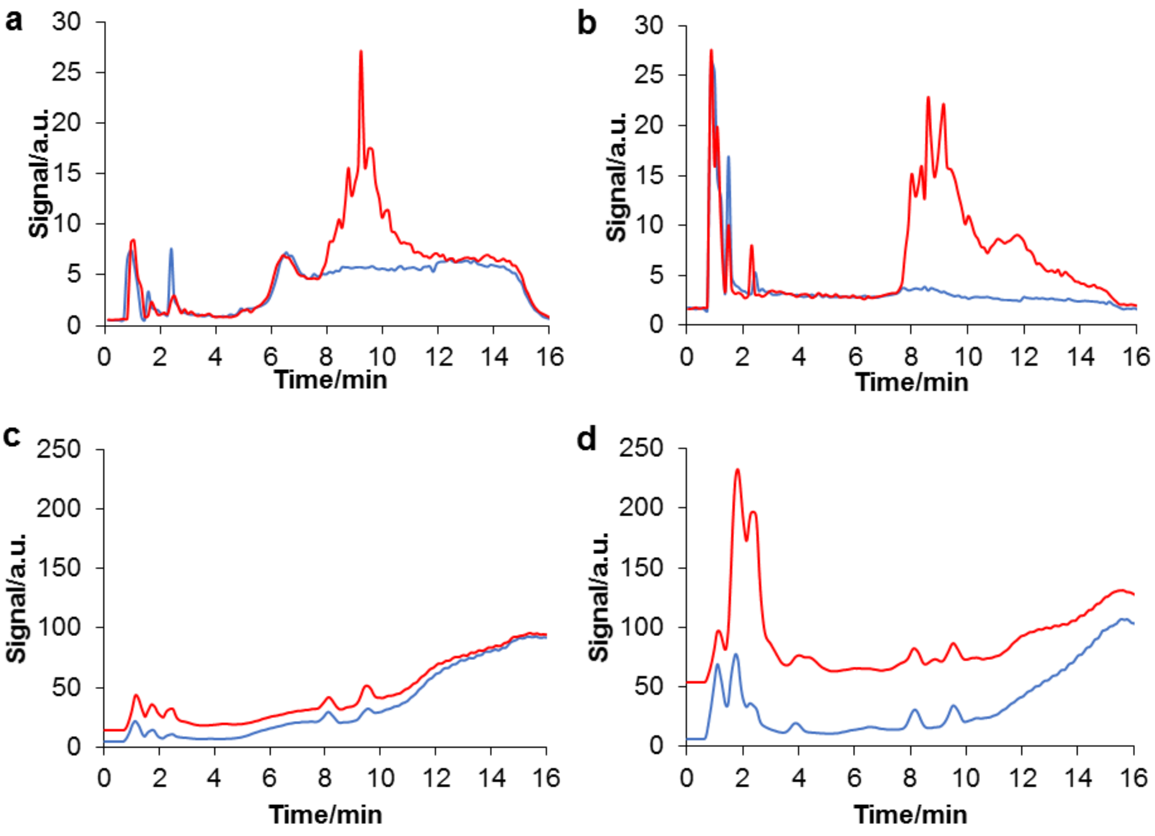

Supplementary material 4. Full scan LC-MS profiles of water extracts in males in (**a)** positive polarity and (**b)** negative polarity, and in females in (**c)** positive polarity and (**d)** negative polarity before (blue) and after (red) spawning.

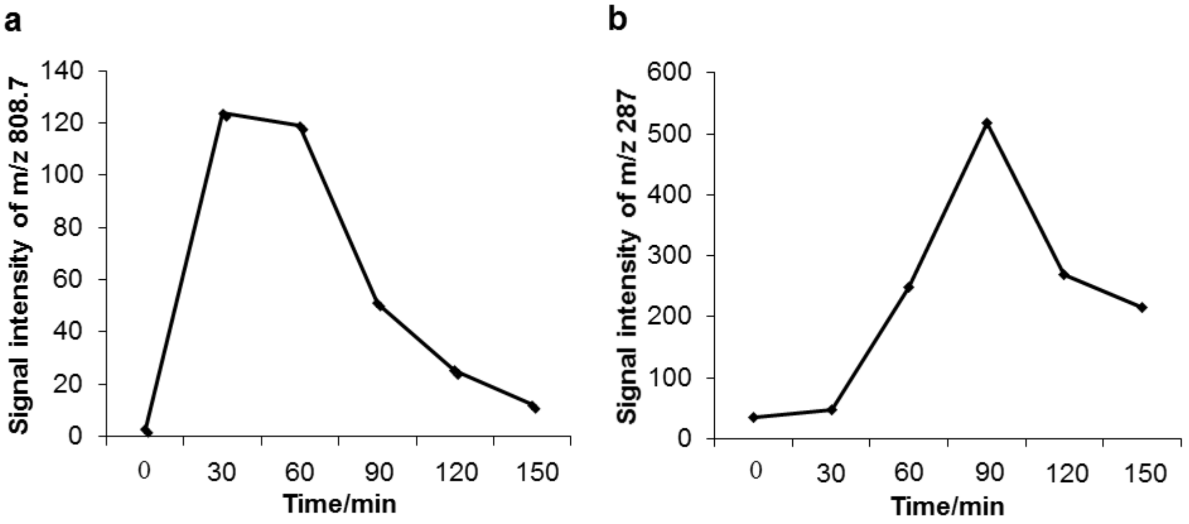

Supplementary material 5. Signal intensity of (**a)** *m/z* 808.7 and (**b)** *m/z* 287 obtained in consecutive extractions of water after spawning.
